# In Vitro Human Microbiota Response to Exposure to Silver Nanoparticles Biosynthesized with Mushroom Extract

**DOI:** 10.3390/nu10050607

**Published:** 2018-05-14

**Authors:** Emanuel Vamanu, Mihaela Ene, Bogdan Biță, Cristina Ionescu, Liviu Crăciun, Ionela Sârbu

**Affiliations:** 1 Faculty of Biotechnology, University of Agronomic Sciences and Veterinary Medicine, Bd. Mărăşti no. 59, district 1, 011464 Bucharest, Romania; 2 Horia Hulubei National Institute for Physics and Nuclear Engineering, 30 Reactorului, 077125 Magurele, Romania; mene@nipne.ro (M.E.); cristina.ionescu@nipne.ro (C.I.); cliviu@nipne.ro (L.C.); 3 Faculty of Physics, University of Bucharest, 405 Atomistilor Blv., 077125 Magurele, Romania; bogdan.bita@imt.ro; 4 ICUB-Research Institute of the University of Bucharest 36-46 Bd. M. Kogalniceanu, 5th District, 050107 Bucharest, Romania; ionela24avram@yahoo.com

**Keywords:** mushroom, scavenging activity, simulation, *Bifidobacterium*, homogentisic acid

## Abstract

The ability to orally administer silver nanoparticles (AgNPs) in enteric capsules implies a direct interaction with the colon microbiota. The in vitro effect provides a portrayal of the functional properties under *in vivo* conditions. The purpose of this study was to describe a green AgNP synthesis process, using aqueous extract from *Lactarius piperatus* mushroom, and to characterize the nanomaterial. We determined its antimicrobial and antioxidant effects *in vitro* in the microbiota of healthy individuals via the GIS1 system—a colon transit simulator. Per the quantitative polymerase chain reaction (qPCR) results, the antimicrobial properties of the AgNPs affected the initial share of different enteric species by decreasing the *Bacteroides*, *Enterobacteriaceae*, and *Lactobacillus* populations and favoring the *Bifidobacterium* group. The association between AgNPs and wild mushroom *L. piperatus* extract had a synergistic antibacterial activity against various pathogenic microorganisms while the mushroom extract reduced biofilm formation. Administration of AgNP maintained its constant antioxidant status, and it was correlated with a reduction in ammonium compounds. The physicochemical characterization of these NPs complemented their biochemical characterization. The maximum ultraviolet-visible spectroscopy (UV-VIS) absorbance was observed at 440 nm, while the Fourier transform infrared (FT-IR) spectrum reached a peak at 3296 cm^–1^, which was correlated with the high-performance liquid chromatographic analysis (HPLC). The major phenolic compound was homogentisic acid. The size (49 ± 16 nm in diameter) and spherical shape of the NPs were correlated with their biological effects *in vitro*.

## 1. Introduction

Noble metal-based nanoparticles (NPs) are widely used in biopharmaceutical applications [1] to develop innovative pharmaceutical forms. Silver NP (AgNP) synthesis, in particular, which uses plant extract as a reducing agent, is more advantageous over any other methods that use microorganisms; this process has received great attention in recent years [2]. Due to its natural antimicrobial properties, silver nanomaterials are largely used in applications that require pathogenic germ inhibition.

Green NP synthesis is a highly effective method for developing a rapid, clean, nontoxic, and eco-friendly technology [3], as it offers the benefit of pH and temperature controls to obtain various structures that aim to deliver bioactive compounds (phenolics, flavonoids, tocopherols, etc.). The novel structure can start from different extracts (aqueous or alcoholic), using the reducing potential of different medicinal plants [3], medicinal mushrooms [4], or bacteria [5]. A recent study showed that aqueous extract from *Pleurotus* (*P.*) *ostreatus* can act as an efficient reducing agent for the large-scale production of novel antimicrobial compounds [6]. Furthermore, extracts from *P. florida* mushrooms represent another example where NPs that have antitumoral effects against a few cancer cell lines were produced [7]. Bioactive compounds in edible mushrooms have high biological value [8], and this may add or bring potentiating effects to the antimicrobial properties of silver. Of all the extracted compounds, polyphenol carboxylic acids are supposed to be responsible for triggering the biological reduction of silver [3]. It is reasonable to assume that the major compound or compounds are responsible for this process. Thus, it is very useful to have the structural details of the compound’s nanostructure, its expected shape, and its direct influence on the biological response.

Thus, this work aimed to evaluate the physical and biochemical properties of AgNPs that were synthesized using an aqueous extract of the *Lactarius* (*L*.) *deliciosus* mushroom as a reducing agent. Nanostructure formation started with AgNO_3_, lasted 24 h, and was completed with an atomization process in a maltodextrin carrier. The *in vitro* antioxidant effects, as well as selective antimicrobial properties, were determined on the microbiota of healthy individuals following a restoration process, using the unicameral GIS1 colon simulator (www.gissystems.ro). Some key metabolic responses (lactate and ammonia production) of the treated microbial communities were analyzed. The NPs were visualized under electronic microscopy (scanning electron microscopy (SEM)), and their ultraviolet visible (UV-Vis) and Fourier transform infrared (FTIR) spectra were determined.

To our knowledge, this is the first report to examine the effect of AgNPs against some bacteria that are part of the intestinal microbiota, in terms of both metabolic and biodiversity changes.

## 2. Materials and Methods

### 2.1. Chemicals

All reagents were purchased from Sigma-Aldrich (St. Louis, MO, USA) in analytical grade.

### 2.2. Mushrooms, Extracts, and Obtaining NPs

Wild edible mushrooms, *L. deliciosus*, were harvested from a resinous forest in Prahova County, Romania. Clean, mature mushrooms were dried in a Memmert Universal heating oven at 45 °C for 24–36 h.

Whole dried mushrooms were chopped into small pieces with a Grindomix Retch (type GM); 200.60 g of dried material was added to 300 mL of sterile distilled water and it was subsequently stirred on a magnetic heating plate (VELP Scientifica, Usmate, Italy) at 60 °C for 1 h. The mixture was cooled and filtered through Whatman No. 1 filter paper under vacuum [9].

AgNP biosynthesis was achieved by mixing 270 mL of 10 mM AgNO_3_ solution with 30 mL of mushroom extract in Duran borosilicate glass bottles. The reduction reaction was carried out at room temperature (23 °C–25 °C) for 24 h [4]. After that, 70 mg/mL of maltodextrin solution was mixed and spray dried using a GEA Niro PRODUCTION MINORTM Spray Dryer (Dusseldorf, Germany). The process yielded a dried brown powder that was filled into capsules (coated with hard gelatin) with a manual capsule-filling machine (for laboratory use). The average weight of the capsules filled with the nanomaterial was 396 ± 22.90 mg.

### 2.3. Nanoparticle Characterization

The newly synthesized nanomaterial was analyzed via UV-VIS by periodic (30-day) readings at 340–700 nm using a Camspec M105 spectrophotometer.

The morphology of the AgNPs was determined and their diameter was estimated by Atomic force microscopy (AFM) (Multi-Mode Nano Scope III a Controller; Digital Instruments Veeco Metrology Group, Santa Barbara, CA, USA) and by Scanning Electron Microscopy (SEM) analysis (NovaTM NanoSEM630; FEI Co., Hillsboro, OR, USA) after washing out the maltodextrin carrier. The washing protocol was similar for the two microscopy techniques: ~200 mg of atomized powder was suspended in 2 mL of deionized water and the sugar enabled dissolving. The colloidal material was pelleted by centrifugation (10,000× *g* for 5 min) and resuspended in fresh water for three times, with vigorous vortexing for 15 min each session; a few drops from the last portion of the suspension were then dried directly on the examination surface and the resultant residue was analyzed under microscope.

For AFM, the sample was allowed to dry free on a Muscovite mica-V support, while, for SEM, the sample was dried on double-sided tape and sprayed with liquid nitrogen prior to examination.

The nanomaterial composition was analyzed by FTIR spectroscopy (Nicolet iS 50 FT-IR Spectrometer; Thermo Fisher Scientific, Waltham, MA, USA) at 380–4000 cm^–1^ spectral resolution in KBr pellets [7].

X-ray diffraction (XRD) analysis was performed using a Shimadzu XRD 6000 diffractometer (Shimadzu Corporation, Kyoto, Japan) at room temperature on the powdered nanomaterials [10].

### 2.4. In Vitro Colon-Simulation Tests

All of the in vitro tests were performed in a one-chamber GIS1 simulator; this was accomplished via a Phase 2 transit simulation through the colon in the uni-compartmental system (http://gissystems.ro/gis-technology/). Reconstitution of the normal microbiota was performed after a mean interval of 7–10 days and it followed the previously described protocol [11]. Microbial communities were sampled from a minimum number of three clinically healthy adult volunteers (Collection No. 1418/23.XI.2017; www.colhumb.com) who had not been treated with antibiotics or any other interfering drugs over the past six months, as these may alter the normal microbiota. To test the effect of the NPs on microbial community evolution, two identical series of treatments were performed by adding one dose (capsule) every 12 h to the in vitro simulator.

### 2.5. Antioxidant Activity

DPPH scavenging activity and reducing power were used to test the antioxidant activity of the NPs, as described previously [12,13]. Inhibition of erythrocyte hemolysis assay [14], a biologically relevant oxidation system, was used to modulate the risk factors for *in vivo* response [15]. Blood samples were obtained from the volunteers (men and women) who had also provided the fecal samples used in this study.

### 2.6. Metabolic Activities of the Microbiota Simulated in the In Vitro GIS1 System

Lactate and ammonia concentrations in treated and untreated microbiota were determined after a seven-day interval from treatment end using enzymatic assays (Lactate Assay Kit and Ammonia Assay Kit, respectively; Sigma-Aldrich Co., St. Louis, MO, USA), which was achieved by reading the color change at 570 nm and 340 nm, respectively, per the manufacturer’s instructions [11].

### 2.7. Microbial Community Evolution by qPCR Analysis

The microbial community was analyzed after the samples were passed through all segments of the GIS1 colon simulator. T0 represents the microbiota at the initial time (no treatment). For each sample, four bacterial groups were quantified in the specific microbiota: the *Lactobacillus* (*L*.) genus (facultative or microaerophilic anaerobes), the *Bacteroides* (*B*.) group (obligate anaerobes), the *Bifidobacterium* (*B*.) genus (anaerobes), and the *Enterobacteriaceae* family (aerobes and facultative anaerobes, including some human pathogens).

DNA was extracted using the Isolate Faecal DNA kit (Bioline, London, UK) and the concentration and purity of DNA was measured using a NanoVue Plus spectrophotometer (GE, Boston, MA, USA).

Quantitative polymerase chain reaction (qPCR) analysis was carried out on a 7900 real-time PCR machine (Applied Biosystems, Foster City, CA, USA) using the Power SYBR Green PCR Master Mix (Applied Biosystems) and 50 ng of DNA template per reaction. The primer sequence (Tm = 60 °C) is given in the Supplementary Materials [16]. For bacterial quantification, standard curves were developed using serial dilutions of a known DNA concentration corresponding to *Escherichia coli* (American Type Culture Collection (ATCC) 10536), *L. plantarum* (ATCC 8014), *B. breve* (ATCC 15700), and *B. fragilis* (DSM 2151). The copy number of the genes was calculated based on the following formula:

number of copies = (DNA amount (ng) × 6.022 × 10^23^)/(genome length (pb) × 1 × 10^9^ × 650)

The results were divided by the number of the 16S gene copies found on each genome of each microbial strain, according to the National Center for Biotechnology Information (NCBI) database. A bacterial universal primer pair was used to determine the bacterial load from each sample. All samples were run in triplicate.

### 2.8. The Antimicrobial Assays and Biofilm Inhibition of AgNPs

The antimicrobial assay was performed in 96-well plates according to CLSI M07-A10 recommendation. The minimal inhibitory concentration (MIC) of the new product was determined against *Staphylococcus aureus* ATCC 6538, *E. coli* ATCC 25922, *Candida albicans* ATCC 10231, as reference strains. Serial two-fold dilutions were performed in a 100 µL volume of broth (MH for bacteria and RPMI 1640 with 0.165M MOPS, 0.03% l-glutamine for yeast strain). Each well was seeded with 10 µL of microbial inoculums with 5 × 10^6^ CFU/mL for bacteria and 5 × 10^3^ CFU/mL for yeast strain. The plates were incubated for 20 h at 37 °C, and MIC values were considered the concentration in which there was observed an over 80% reduction in growth compared with the control [17]. After MIC was determined, the plates were washed three times with sterile distilled water and dye with 1% crystal violet in methanol for 15 min. The plates were washed, the dye was extracted with 33% acetic acid and the absorbance at 490 nm was measured by Synergy HTX (Biotek, Winooski, VT, USA) to determine the minimum biofilm eradication concentrations (MBEC) [18].

### 2.9. Statistical Analysis

All parameters were assessed in triplicate, and the results are expressed as the means ± standard deviations, as calculated with the Microsoft Excel software program (Microsoft Office 2010 package; Microsoft Corporation, Redmond, WA, USA). A correlation analysis was performed with GraphPad Prism 6.0 (GraphPad Software Inc., La Jolla, CA, USA). Significance was set at *p* ≤ 0.05.

## 3. Results

### 3.1. AgNP Characterization

**Figure 1:**
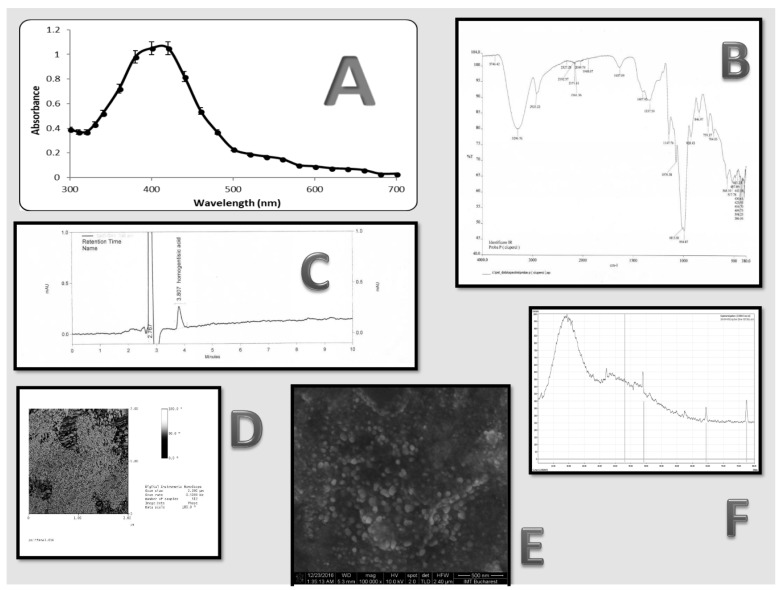
Characterization of AgNPs: (**A**) Ultraviolet-visible (UV-VIS) spectrum; (**B**) Fourier transform infrared (FTIR) spectrum; (**C**) High-performance liquid chromatographic (HPLC) analysis; (**D**) Atomic force microscopy (AFM) image; (**E**) Scanning electron microscopy (SEM) image; and (**F**) X-ray diffraction (XRD) image.

A reduction of Ag^+^ ions in aqueous extract and NP synthesis was marked by color changes in the reaction mix, which ranged from colorless to reddish brown. The maximum UV-VIS absorbance was reached at 440 nm (Figure 1A), regardless of the reaction time. The peaks were influenced by various factors such as size, shape, and particle formulation [19].

Similar to what was reported in a recent study [20], the FTIR spectrum (Figure 1B) indicated a strong peak at 3296 cm^–1^ (phenolic –OH group stretching) and 2925 cm^–1^ (aromatic C–H group stretching and presence of -CH groups). The bond responsible for NP synthesis due to Ag reduction was identified at 1147.76 cm^−1^ which correspond with the FTIR spectra of the compound [21]. Compared to published studies [22,23] of FTIR measurement, we could determine medium intense bands at 1407 and 1637 cm^−1^ characteristic of the N–H amide group and C=O amine group, respectively, which correspond with the presence of protein. These data, showing proteins as capping agent for AgNPs, are correlated with the known composition of *L. piperatus* and increase the stability of the nanoparticles in this case. For silver metal, we identified a strong peak at 386 cm^−1^.

Further, similar to another prior study [24], these NPs presented with a high degree of stability in aqueous solution, neither demonstrating aggregation nor disintegration after 12 months at room temperature. This stability was also maintained while the NPs were in dried form, with a maltodextrin carrier, following a spray-drying process (data not shown).

In such bio-reduction processes, the main secondary products are polyphenol-carboxylic acids [2]. The mixture that resulted from the atomization was confirmed in a previous study [25]—the chromatogram showed a significant amount of homogentisic acid, which putatively acted as a single reducing acid, an aspect proved by the FTIR spectra (Figure 1C). This does not exclude the presence of minute amounts of other bioactive compounds (e.g., alkaloids) [26], as shown by the high number of peaks on the FTIR spectra. This did not influence nanomaterial biosynthesis.

**Figure 2:**
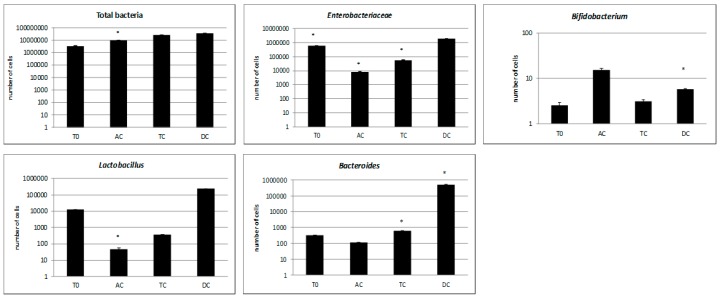
The numbers of the main bacterial groups detected after the AgNP treatment. For each sample, the minimum number of replicates was *n* = 3. The stars indicate significant enrichment (*p* < 0.05). T0: restored microbiota, AC: ascending colon, TC: transverse colon, DC: descending colon.

AgNPs synthesized with *L. deliciosus* extract presented with high vibration stretches for the two aforementioned chemical bonds (Figure 1B) of the major polyphenolic acid (homogentisic acid; Figure 2). This bioactive compound was acting as both capping and stabilizing agent, which is similar to what was reported for studies on *Artocarpus altilis* [24].

SEM analysis showed the presence of spherical NPs that ranged in size from 33 to 64 nm, with an average diameter of 49 ± 16 nm. The results were confirmed with AFM (Figure 1D); here, the NPs demonstrated high variability, which was likely influenced by the heterogeneity of macromolecular complexes of the *L. piperatus* extracts. Similar sizes were obtained in previous published data [6,24]. The SEM examination (Figure 1E) showed a homogeneous particle distribution with a roughly spherical geometry. The various sizes may result from the different time intervals when reduction was accomplished, which is in accordance with a previous study that was performed with cell-free supernatants of *Acinetobacter baumannii* [27]. The sizes and shapes of metal NPs are affected by various factors, including pH, time of incubation, precursor concentration, reductant concentration, temperature, and method of preparation [24].

The XRD analysis was performed to confirm the crystalline nature of the AgNPs. A typical XRD pattern was determined with five intense peaks ranging from 300 to 800 (Figure 1F). The explicit 2 theta peak values of 33°, 44.5°, 65°, and 77° were determined and correspond with standard silver values, similar with previously data [24]. An analysis of the XRD spectrum with the standard and previous study confirmed that the silver particles took on the form of nanocrystals, and silver was present [22,28]. Another significant result that emerged from the XRD analysis was the presence of a peak at around 32°, which was similar to another phase (likely, Ag_2_O). This is not characteristic of bioorganic compounds. This result could appear spontaneously during the measurement under air in the nanostructure powder. The XRD analysis was sustained by the previous research [29] where silver nanoparticles were synthesized from red algae, *Gracilaria crassa*.

### 3.2. Microbiological Analysis of the Microbiota Composition. Approach of Antimicrobial Effect and Biofilm Inhibition

The AgNPs exerted a higher antimicrobial activity against all microbial strains compared with the AgNO_3_ and wild mushroom *L. piperatus* extract, which also had antimicrobial effect, especially against *C. albicans* and *E. coli* (Table 1). The AgNPs and wild mushroom *L. piperatus* extract association had a synergistic antibacterial activity against a wide range of microbial strains. The AgNO_3_ and AgNPs did not have any antibiofilm effect on bacterial strains because the MBEC values corresponded with MIC values, so the biofilm eradication was caused by the antimicrobial activity. AgNPs slightly stimulated *C. albicans* biofilm formation.

Instead, wild mushroom *L. piperatus* extract had antibiofilm effect on all microbial strains: the MBEC values were under MIC values, so the biofilm reduction could not be associated with antimicrobial activity exerted by bioactive compounds from mushroom extract (Table 2).

The DNA used for qPCR (0.11–1.09 µg/µL) was free of protein contamination, with an A_260_/A_280_ ratio between 1.70 and 1.82.

The bacterial load of the sampled segments was approximately similar, around 10^7^ cells/mL, except for the initial samples (T0), where the number of cells was slightly lower. In the first segment of the colon (ascending colon (AC)), the AgNPs demonstrated an antimicrobial effect against the main bacterial groups (*Bacteroides*, *Enterobacteriaceae*, and *Lactobacillus*), while the *Bifidobacterium* population increased due to either a lack of sensitivity or to the initial decline in their large number and competitiveness. The *Enterobacteriaceae* group and *Lactobacillus* species appeared to be most susceptible to the AgNPs, as their populations decreased more than 100 times after treatment. Among the affected groups, the *Bacteroides* species proved the most resistant to the AgNPs, as their population decreased less than 10 times.

While passing through the GIS system, the cell concentration of the *Bacteroides*, *Enterobacteriaceae*, and *Lactobacillus* populations greatly increased—a remarkable occurrence for the lactobacilli, which increased from 10^2^ cells/mL to 10^5^ cells/mL (Figure 2).

### 3.3. Antioxidant and Metabolic Capacity after AgNP Administration

**Figure 3:**
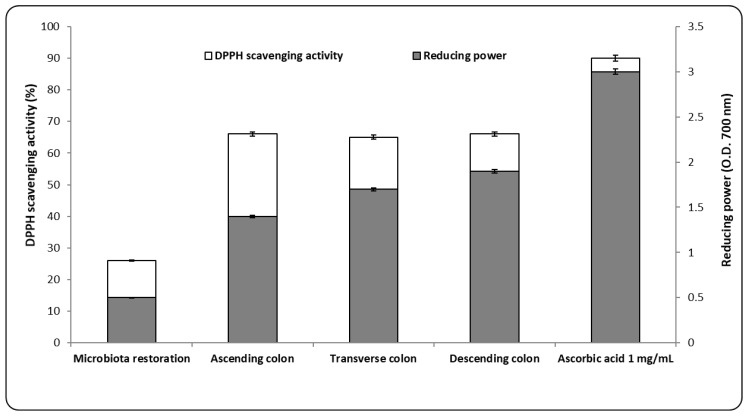
DPPH scavenging activity and reducing power following AgNP treatment. For each sample, the minimum number of replicates was *n* = 3 (*p* < 0.05).

The antioxidant activity proved by the scavenging activity of DPPH had a constant value (65 ± 0.50%) for each colon segment. The value was 23.52 ± 0.09% lower than the control. The percentage was approximately 60% higher than that determined following microbiota restoration (Figure 3).

A similar trend was also calculated for the reduction power (Figure 3). The difference between the value of the restored microbiota and the administration of the AgNPs was, on average, 60% as well. These results demonstrated that the nanomaterial acted as an antioxidant, which was due to the homogentisic acid present in the administered powder. Similar to what was reported in previous studies, these bioactive compounds that were involved in obtaining the AgNPs increased their redox potential, leading to the effect where free radicals exhibited scavenging activity and were bound to the metal ions responsible for free radical formation [30].

Another analysis determined the antioxidant potential from colon segments by *ex vivo* tests. From one segment to other, the level of inhibition of erythrocyte hemolysis was higher, by no more than 20% (data not show, but the effect was based on the presence of homogentisic acid in the *in vitro* environment. The low level obtained after *ex vivo* tests was interpreted as the limitative effects of AgNPs, which promoted hemolysis [31].

The metabolic signature determined after the administration of the AgNPs was identical to their antioxidant profile. The loss of a favorable component led to a high accumulation of ammonia when compared with the restored microbiome. The significant difference was up to 50% at the level of the descending segment, where it had a reached maximum of 200 mg/L. No significant variation in lactate levels was identified, which is mostly associated with the presence of *Lactobacillus* strains. The results were indicative of a type of compensation that occurred in response to the significant presence of the Bifidobacterium strains. Overall, the presence of the two microbial metabolism products was considered positively correlated (R^2^ = 0.711; *p* < 0.01), which both confirmed the findings of previous studies [32] and marked the result of AgNP action.

## 4. Discussion

Although the structure of the human microbiome is affected by the administration of AgNPs, green synthesis mediated by mushroom extract does not affect the microbiome’s in vitro fingerprint. This study confirms that only minor microbial alterations in the human microbiota occurred, proving the importance of the phenolic component of the AgNP structure (Figure 1C) in maintaining the balance in the human microbiome. No correlation was identified for any alterations in microbial metabolism [11]; however, this does not exclude the silver ion toxicity that occurs in humans following long-term administration [33]. The results suggest the antimicrobial significance of Ag(0) NP oxidation by O_2_ in the antibacterial activity, through the release of Ag^+^ [34]. This point of view is sustained by the constructive capacity of the GIS1 *in vitro* system, which is not a full anaerobic system.

The presence of AgNPs was positively correlated with the presence of *Bacteroides*, an anaerobic representative, likely due to a higher silver ion tolerance, which guarantees their persistence. The reduction of silver ions with homogentisic acid determined a distinct biological response, which is correlated with particle size and an irregular oval shape. Gram-negative strains are more sensitive in this state. The homogentisic acid was present *in vitro* in all segments, which generated a positive correlation (R^2^ > 0.7) with the presence of beneficial microbial strains. The moderate administration of AgNPs, combined with a biologically active compound, has resulted in selective susceptibility to the action of AgNPs [32], which corresponds to a new biologic response toward biocompatible nanomaterial administration.

Revealing the relationship between the type of nanomaterial, the biological response (*in vitro* and *ex vivo*), and the prevalent genres in the microbiota has improved our understanding of AgNPs’ contribution toward modifying the microbiome structure. In the tested microbiota, this biological effect was completed when there was a moderate increase in free-chain inhibition capacity, which corresponded to a maximum DPPH scavenging activity of 66 ± 0.80%. It was 7% higher, on average, than the inhibitory capacity of the same radical in the restored microbiota. The same profile was also determined for reduction power and the values were about 20% lower than for the 1% ascorbic acid.

Unlike the previous study, the presence of the biochemically synthesized nanomaterial in the colon did not yield a positive correlation between its antioxidant potential and favorable strains [35]. Otherwise, a low correlation between scavenging activity and *ex vivo* test was determined (R^2^ < 0.15, *p* < 0.03) which was a response of high hemolysis capacity of AgNPs. Presence of biologically active molecules could decrease the *in vivo* biological toxicity. This impact on biological material was proved by other tests where the nanosamples had significantly higher hemolytic rates if the size of the AgNPs was of maximum 100 nm [36]. Our AgNPs was positively correlated with size and biological responses *in vitro* and *ex vivo*. As was the case during the consumption of functional food (edible wild mushrooms), AgNPs generated a reduction in ammonium synthesis with a measured level below 100 μg/mL, which is 20% lower than in the restored microbiota.

In the tested samples, the ascending colon was the segment most affected by the presence of nanomaterial, which featured a highly decreasing number of lactic acid bacteria; conversely, the response of the free radical was not depleted. This behavior can be attributed to homogentisic acid because it was the only active compound present in the in vitro environment with a high antimicrobial effect [37]. It is plausible that the reduction of favorable strains in the first phase of the *in vitro* test resulted from the combined effect of silver ions and phenolic acid, which may account for an imbalance in the synthesis and accumulation of microbial metabolites. This triggered the increase of *Bifidobacterium* strains in the same segment (Figure 2). The impact is countervailed during transit by stable microbiota. Conversely, the impact will be profoundly negative in cases of imbalanced microbiota, as is found in those who are nutritionally ill (e.g., diabetics) [11].

Thus, the results of the study prove the possibility to use such nanomaterials on people affected by pathologies associated with disturbed microbiota. It also shows limited effects in the interaction between nanomaterial and epithelial cells in the colon, and it further illustrates their influence on microbial adherence. *In vitro*, the tested AgNPs did not significantly affect biofilm formation on the surface of light glass balls on the bottom of the simulation vessel. This statement sustains the importance of biosynthetic route of AgNPs and proves the biological effects of sublethal concentration of this functional material. The results were sustained by the results presented in Table 1 and Table 2 and proved the positive functional effects in the disturbed microbiota of target groups. The results were correlated with previous studies where a biosynthetic method to obtain AgNPs with leaf extract of *Allophylus cobbe* was used [38].

## 5. Conclusions

To conclude, AgNPs were synthesized beginning with an aqueous extract of the *L. deliciosus* mushroom and it was characterized by UV-VIS spectroscopy, FTIR, SEM, and XRD analysis. qPCR analysis revealed that the nanomaterial directly impacted the human microbiota’s composition, which highlights its potential for novel applications in cases of controlled ingestion. Here, we demonstrated the lack of interference of AgNPs with the *Bifidobacterium* population, which was correlated with a constant antioxidant capacity in the simulated colon regions. Thus, the green biosynthesis of AgNPs, as based on mushroom extract, could be used in the perturbed microbiota and response to oxidative stress which is strongly correlated in these degenerative pathologies.

## Figures and Tables

**Table 1:** The MIC values of the tested compounds.

Samples	*Staphylococcus aureus*	*Escherichia coli*	*Candida albicans*
**AgNO_3_ (1 mM)**	250 µM	62.5 µM	7.8 µM
**Extract (10% *v*/*v*)**	2.5%	2.5%	1.25%
**AgNPs (200 mg/mL)**	1.56 mg/mL	0.39 mg/mL	˂0.098 mg/mL

**Table 2:** The MBEC values of the tested compounds.

Samples	*Staphylococcus aureus*	*Escherichia coli*	*Candida albicans*
**AgNO_3_**	250 µM	62.5 µM	7.8 µM
**Extract**	0.312%	0.156%	˂0.0045%
**AgNPs**	1.562 mg/mL	0.39 mg/mL	50 mg/mL
